# Apoptotic M540 bodies present in human semen interfere with flow cytometry-assisted assessment of sperm DNA fragmentation and oxidation

**DOI:** 10.1186/s12610-021-00143-7

**Published:** 2021-10-21

**Authors:** Niloofar Sadeghi, Marziyeh Tavalaee, Abbas Kiani-Esfahani, Aron Moazamian, Parviz Gharagozloo, Robert J. Aitken, Joël R. Drevet, Mohammad Hossein Nasr-Esfahani

**Affiliations:** 1 Department of Animal Biotechnology, Reproductive Biomedicine Research Center, Royan Institute for Biotechnology, ACECR, Isfahan, Iran; 2 CelloXess LLC, 830 Bear Tavern Road, Ewing, NJ 08628 USA; 3 Priority Research Centre for Reproductive Sciences, Faculty of Science and Faculty of Health and Medicine, The University of Newcastle, Callaghan, NSW 2308 Australia; 4 Hunter Medical Research Institute, New Lambton Heights, NSW 2305 Australia; 5 GReD Institute, CRBC, Faculté de Médecine, INSERM U1103-CNRS UMR6293-Université Clermont Auvergne, 28 Place Henri-Dunant, 63000 Clermont-Ferrand, France; 6 Isfahan Fertility and Infertility Center, Isfahan, Iran

**Keywords:** Spermatozoa, TUNEL, SCSA, 8-OHdG, Merocyanine 540, Non-cellular structures, Sperm nuclear damage, spermatozoïdes, TUNEL, SCSA, 8-OHdG, mérocyanine 540, structures non cellulaires, dommages nucléaires aux spermatozoïdes

## Abstract

**Background:**

The use of flow cytometry (FC) to evaluate sperm DNA fragmentation via deoxynucleotidyl transferase terminal fluorescein dUTP nick-end labeling (TUNEL) has shown inconsistencies compared with conventional fluorescent microscopic analyses. It has been hypothesized that the observed discrepancies could be attributed to the presence of apoptotic bodies that can be labeled with merocyanine 540, the so-called M540 bodies. In order to verify this hypothesis and determine the accuracy of our in-house FC-assisted evaluation of spermatozoa parameters, we used FC to evaluate both the fragmentation of sperm DNA using the TUNEL assay and the oxidation of sperm DNA using the 8-OHdG assay on semen samples with or without M540 bodies.

**Results:**

We show that the presence of M540 bodies lead to underestimation of both the level of sperm DNA fragmentation and sperm DNA oxidation when using FC assisted detection systems. We also observed that this situation is particularly pertinent in semen samples classified as abnormal with respect to the routine WHO semen evaluation as they appear to contain more M540 bodies than normal samples.

**Conclusions:**

We conclude that M540 bodies interfere with both FC-conducted assays designed to evaluate sperm nuclear/DNA integrity. Exclusion of these contaminants in unprepared semen samples should be performed in order to correctly appreciate the true level of sperm DNA/nuclear damage which is known to be a critical male factor for reproductive success.

**Supplementary Information:**

The online version contains supplementary material available at 10.1186/s12610-021-00143-7.

## Résumé

**Contexte:**

L’utilisation de la cytométrie en flux (CF) pour évaluer la fragmentation de l’ADN des spermatozoïdes via la technique TUNEL (Terminal transferase dUTP nick-end labelling) a montré des incohérences par rapport aux analyses conventionnelles par microscopie fluorescente. L’hypothèse a été émise que les divergences observées pourraient être attribuées à la présence de corps apoptotiques qui peuvent être marqués à la mérocyanine 540 (corps M540). Afin de vérifier cette hypothèse et de déterminer la précision de notre évaluation interne des paramètres des spermatozoïdes, nous avons mesuré par CF à la fois la fragmentation de l’ADN des spermatozoïdes en utilisant le test TUNEL et l’oxydation de l’ADN des spermatozoïdes en utilisant le test 8-OHdG sur des échantillons de sperme avec ou sans corps M540.

**Résultats:**

Nous montrons que la présence des corps M540 entraîne une sous-estimation du niveau de fragmentation et d’oxydation de l’ADN des spermatozoïdes lors de l’utilisation de systèmes de détection assistée par CF. Nous avons également observé que cette situation est exacerbée dans les échantillons de sperme classés comme anormaux (selon les standards de l’OMS), car ces derniers semblent contenir plus de corps M540 que les échantillons normaux.

**Conclusions:**

Nous concluons que les corps M540 interfèrent avec les deux tests conduits par CF et conçus pour évaluer l’intégrité nucléaire des spermatozoïdes. L’exclusion de ces contaminants dans les échantillons de sperme non préparés devrait être considérée afin d’apprécier correctement le véritable niveau de dommages au noyau spermatique qui est connu pour être un facteur critique pour le succès reproductif.

## Background

With the advent of assisted reproductive technologies (ART), it is becoming increasingly clear that reproductive success is intimately linked to the integrity of both gametes. From the male perspective, there is now a good consensus that the standard seminal evaluation recommended by the World Health Organization [1] is no longer sufficient to assess an individual’s potential for successful reproduction. A closer inspection of the sperm cell is required, particularly with regard to the integrity of the paternal nucleus and DNA. Over the past ten years, scientists and clinicians have agreed that condensation, fragmentation and, more recently, oxidation of the sperm nucleus are important parameters that determine the success of reproduction which go beyond fertilization success rate. This is particularly relevant when considering the fidelity of embryonic development and the quality of life of the offspring. Even though it is not yet routinely examined, infertility clinicians have now reached a consensus on the fact that sperm nuclear/DNA damage affects ART outcomes by challenging the oocyte repair capacity [2–5]. Sperm DNA/nuclear damage has far reaching consequences because if for any reason (e.g. extensive alterations to the sperm nucleus or/and a deficient oocyte repair system associated with advanced maternal age) paternal nuclear damage is not fully repaired by the oocyte, it can introduce de novo mutations that will impair embryo development and be transmitted to the next generation potentially affecting offspring quality of life [5–11].

These findings have led to the development of several assays aimed at evaluating the integrity of the sperm nucleus according to its level of condensation, fragmentation and oxidative state. These three conditions are closely related, but can also exist independently of each other [5], further confusing spermatozoa integrity evaluation. Acridine orange (AO) was used as early as the seventies to visualize the integrity of bull spermatozoa [12]. Following on from this, Evenson et al. (1980) developed the sperm chromatin structure assay (SCSA®) [13], which, with time has become one of the most widely used and reliable assays to evaluate the level of compaction and, indirectly, the level of fragmentation in the spermatozoa nucleus. This assay was followed by the TUNEL assay in 1990, the Comet assay in 1996 and the sperm chromatin dispersion assay (SCD) in 2003, all of which claim to somehow evaluate sperm DNA fragmentation (SDF) [14]. Although very different in nature, both the TUNEL and SCSA may use flow cytometry (FC) allowing automated analysis of a large number of cells within a single sample, thereby providing a more accurate and operator-independent evaluation. These advantages make FC-assisted TUNEL and SCSA the tests of choice for clinicians who wish to assess sperm DNA/nuclear integrity as an indicator of risk of reproductive failure [15–17].

**Fig. 1:**
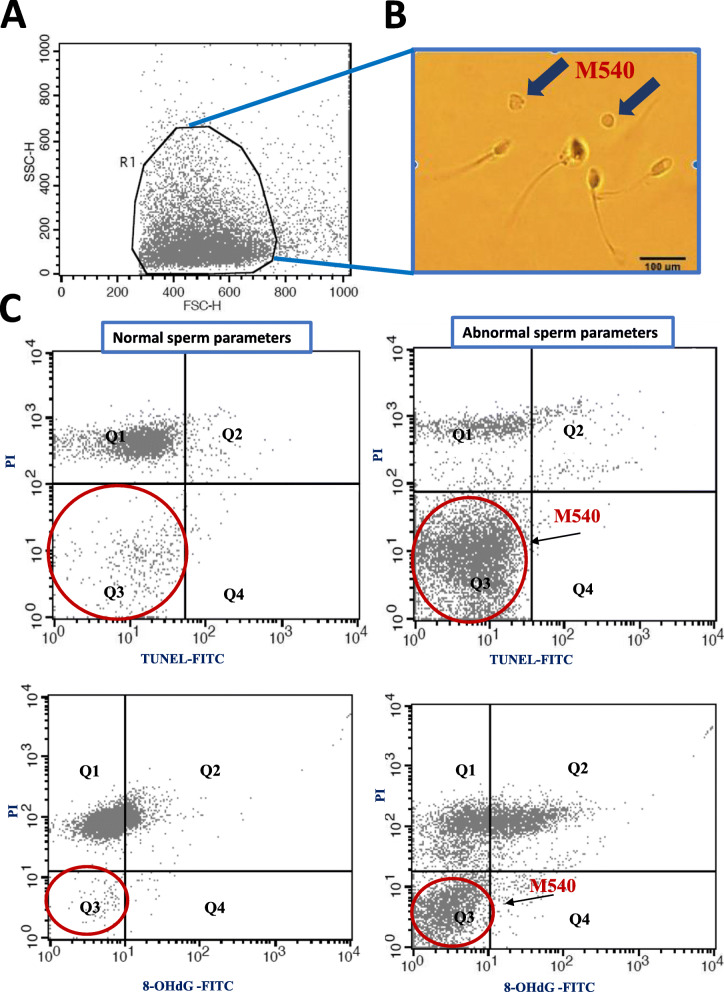
**A**) FSC/SSC dot plot identifying the flame shaped region (R1) containing spermatozoa and M540 bodies. **B**) Representative microscopic photograph showing a semen sample containing spermatozoa and M540 bodies (magnification: × 200). **C)** representative TUNEL and 8-OHdG flow cytometric charts of a sperm sample from the cohort having “normal sperm parameters” (left panels) and a sperm sample from the cohort having “abnormal sperm parameters” (right panels). Q4: TUNEL-8-OHdG positive/PI negative, Q3: PI negative/TUNEL-8-OHdG negative, Q2: PI-positive/TUNEL-8-OHdG positive, Q1: PI-positive/TUNEL-8-OHdG negative. Note: apoptotic or M540 bodies (arrows) appear in Q3

It is not our intention to debate which of these assays (TUNEL versus SCSA) is most trustworthy with regard to assessing sperm DNA/nuclear integrity. What we would like to emphasize is that because both assays use FC, analyses of sperm samples might be compromised by the presence of interfering structures resulting from apoptotic spermatozoa. These structures can be labeled using merocyanine (M540) and as such have been termed M540 bodies [18–20]. With their similarity in size and density to sperm heads (see an example in Fig. 1, panels A & B), these structures have been suspected of interfering with FC evaluation because the flow cytometer is unable to distinguish precisely which structures emit fluorescence [20–22]. This inter-technique lack of concordance when FC data are compared with FM data [17] is a major problem which prevents clinicians from delivering an accurate diagnosis. It also renders inter-clinic comparisons ineffectual partly explaining the low confidence the clinical community shows towards such assays.

In this context, the goal of this study performed in our local infertility clinic, was to assess the impact of M540 bodies on FC used to evaluate the level of sperm DNA fragmentation via the TUNEL assay and the level of sperm DNA oxidation via the 8-OHdG assay.

## Materials & methods

### Sample collection and sperm preparation

The study was approved by the Royan Institute Ethics Board (NO: 98000030). Patients were the male partner of couples entering the Isfahan Fertility and Infertility Center (Isfahan, Iran). Each patient completed a questionnaire pertaining to general health issues and medical history information. Written informed consent was obtained in parallel for each patient. Semen samples (*n* = 180) were collected according to WHO criteria [1] as part of the routine semen analysis at the Isfahan Fertility and Infertility Center. Patients on medication and semen samples with leukocytospermia were excluded from the study because leukocytospermia may affect sperm DNA fragmentation and induce variation between the two groups. After an abstinence period of at least 48 h and a maximum of 6 days, (as recommended by standard WHO2010 procedures), semen samples were obtained by masturbation, collected in sterile containers and allowed to liquefy for a period of 15–30 min at room temperature. Basic semen parameters were evaluated according to WHO recommendations [1]. Semen samples with a sperm cell concentration above 30.10^6^/ml, a volume ≥ 1.5 ml, motility ≥40%, and at least 4% of the cells exhibiting normal morphology were considered normal. Samples that did not meet these thresholds were classified as abnormal [1]. In addition to the standard analysis of sperm parameters, the TUNEL assay and SCSA were performed in order to assess the level of fragmentation of the sperm nucleus. Evaluations were performed on unprepared/unfractionated semen sample in order to gain a picture of overall semen quality. These tests were conducted in 2 sub-cohorts (*n* = 90 each), one comprising normozoospermic patients and the other comprising patients with abnormal standard sperm parameters [1]. Furthermore, an analysis of the level of oxidation of the sperm nucleus by measuring the content of 8-OHdG residues was also performed on 27 samples (13 normozoospermic samples and 14 samples with abnormal standard sperm parameters). Flow cytometry using a FACS-Calibur flow cytometer (Becton Dickinson, San Jose, CA, USA) was selected to carry out the TUNEL, SCSA and 8-OHdG assays as originally described [14, 16, 23], respectively. Propidium iodide (PI) staining was used in parallel to exclude M540 bodies from the FC analyses.

### TUNEL assay

DNA fragmentation was evaluated using a terminal deoxynucleotidyl transferase dUTP nick end labeling (TUNEL).

assay Sperm DNA fragmentation evaluation was performed using a fluorescein apoptosis detection kit (Apoptosis Detection System Fluorescein; Promega, Germany) following the recommendations of the supplier. In brief, the washed semen samples were fixed with 4% paraformaldehyde for 25 min and permeabilized with 0.2% Triton X-100 for 5 min. The cells were then washed twice with 1x PBS and incubated for 1 h at 37 °C in the DNA labeling solution, which includes the reaction buffer, terminal deoxynucleotidyltransferase (TdT) enzyme, and FITC-dUTP provided with the kits. The samples were then centrifuged and the resulting pellets were resuspended in the propidium iodide staining solution (50 μM) for 5 min before undergoing FC analysis. The evaluation of DNA fragmentation was performed as described by the manufacturer. A negative control was prepared for each test sample, omitting the addition of the TdT terminal transferase [14]. As shown in the representative TUNEL plots presented in Fig. 1C, PI-positive cell gating was used to select the sperm population for TUNEL analysis. Since PI selectively detects dead cells, the contaminating apoptotic bodies that did not contain chromatin were unmarked [15]. Therefore, the percentages of TUNEL-positive spermatozoa were obtained by examining cells positive for both PI and FITC [14, 24].

### SCSA

As originally described in Evenson et al., [13], 2 × 10^6^ spermatozoa *per* sample were resuspended in TNE 1× solution to obtain a final volume of 1 ml. Then, 1.2 ml of acridine orange staining solution (Sigma, St. Louis, USA) was mixed with 200 μl of sample (controls) or 400 μl of acid–detergent solution and 200 μl of sample brought together for 30 s (tests). At least 10,000 sperm cells were evaluated to determine the percentage of DNA fragmentation.

### 8-OHdG assay

8-OHdG (or 8-oxodG), an adduct of the oxidized guanine base, was detected to evaluate oxidative damage to sperm DNA. In brief, samples were washed in PBS and the pellet was resuspended in decondensation buffer containing 2 mM dithiothreitol (DTT), 0.5% Triton X-100 and 1× PBS, and incubated for 20 min at 4 °C. After washing with PBS, the samples were fixed in 4% paraformaldehyde for 20 min at 4 °C. The fixed cells were washed and incubated in 3% normal goat serum saturation solution (Chemicon International, ThermoFisher Scientific, Temecula, CA, USA). The samples were then incubated overnight with an anti-8-OHdG monoclonal antibody (NB110–96878, Novus Biologicals, The Netherlands) at a dilution of 1:1500 and, subsequently, with a secondary antibody (Alexa Fluor 488, dilution 1:1500) for 90 min at 4 °C. The samples were washed, resuspended in 500 μl PBS and stained with PI (50 μM in PBS) for 5 min in the dark at room temperature before loading on to the FC. Positive controls were prepared by incubating the samples with H_2_O_2_ (10 mM) for 1 h at room temperature prior to the fixation procedure [23]. As with the TUNEL method, 8-OHdG-positive sperm cell percentages were obtained from the PI/FITC plot as the percentage of spermatozoa that were positive for both PI and FITC [21]. Representative FC plots are presented in Fig. 1.

### Statistical analysis

The Statistical Package for the Social Sciences for Windows, version 18.0 was used for data analysis. Shapiro–Wilk and Levene tests were performed for normal distribution and equal variance, respectively. All results from this study are presented as mean ± standard deviation (SD) for continuous variables and a frequency (percentages) for categorical variables. The T-test for independent samples was used to compare the data between two groups. To find the association between two categorical data, the Chi-square test was then used. Differences were considered statistically significant when *P* < 0.05.

## Results

Standard semen parameters including spermatozoa concentration, motility and morphology were determined to constitute two cohorts (*n* = 90 each; see supplementary figure 1A). These were designated as “normal” (i.e. normozoospermic) and “abnormal” according to WHO standard selection criteria (WHO, 2010 [1]). The percentage of motile spermatozoa (59.07 ± 13.9 vs. 35.10 ± 20; *P* < 0.001), was the most discriminant factor between the two cohorts (normal vs abnormal, respectively). Sperm concentrations were also significantly different between the two cohorts (81.58 ± 43.1 vs. 50.19 ± 42.6, expressed as 10^6^ spermatozoa/ml, normal vs abnormal, respectively) although they remain within the range WHO considers as normal (40 to 300 10^6^ cells/ml). The percentage of sperm cells having an abnormal morphology was not found to differ between the two cohorts (95.05 ± 1.2 vs. 97.06 ± 1.1; normal vs abnormal, respectively).

### Sperm DNA fragmentation analysis

**Fig. 2:**
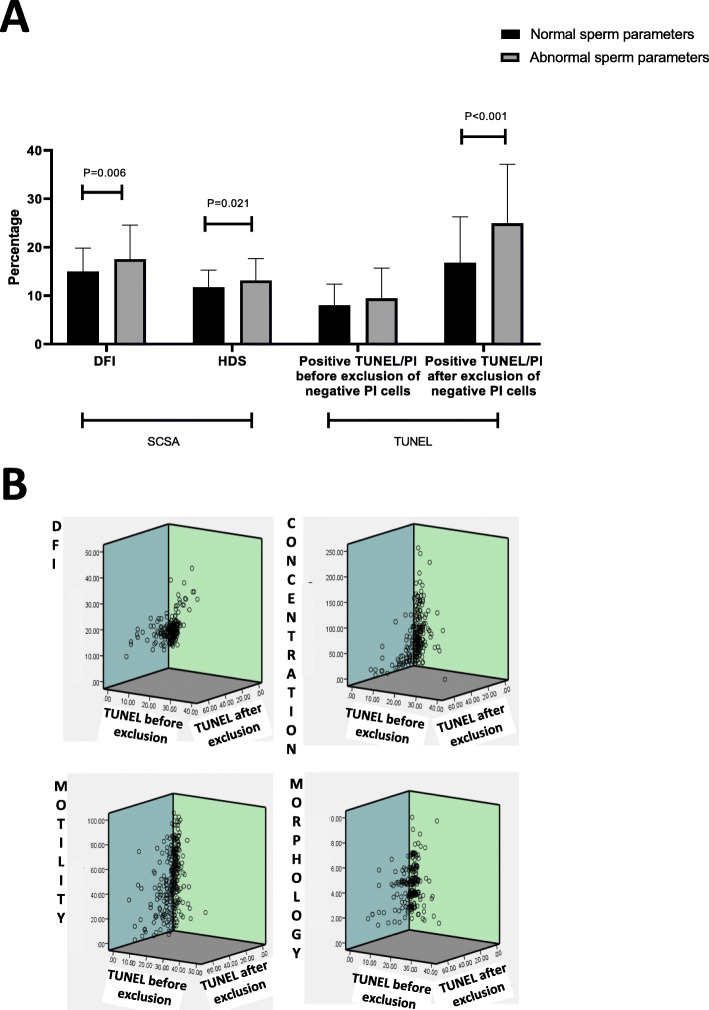
**A)** Comparison of sperm DNA fragmentation between individuals with normal and abnormal sperm parameters. For TUNEL analysis, PI-negative were excluded by excluding Q3 and Q4 quadrant of the flow chart. Data are presented as the mean ± standard deviation. **B)** 3D scatter plots showing sperm DNA fragmentation (DFI) assessed by the TUNEL assay before and after exclusion of unstained cells by PI. If there was no difference, all points should line up on the Y axis. As the scatter for all 4 parameters monitored is evident, this suggests that the presence of M540 bodies is highly likely to influence the parameter measured

As shown in Fig. 2A, the SCSA revealed statistically significant differences between the two sub-cohorts in terms of DNA Fragmentation Index (DFI) and High DNA Stainability (HDS), with the “abnormal” subcohort having higher DFI and HDS indices (*p* = 0.006 and *p* = 0.021, respectively). In contrast, analysis by the TUNEL test (Fig. 2A) revealed no statistically significant difference between the two subcohorts prior to the exclusion of PI-negative cells*.* However, when PI-negative elements were excluded (exclusion of quadrants Q3 and Q4 as shown in Fig. 1), then a statistically significant difference (*P* < 0.001) was observed between the two sub-cohorts with a higher percentage of TUNEL-positive spermatozoa in the “abnormal” sample group (Fig. 2A).

Figure 2B is a dispersion correlation analysis of the entire sample population (*n* = 180) for 4 measured parameters. If the M540 bodies had no effect on the measured parameters, all points (which correspond to the comparison of the measured parameter before or after the exclusion of the M540) seen on the graphs should be aligned on the Y-axis. The observed deviation attests that the measured parameters are somehow influenced by the presence/absence of M540 body.

**Fig. 3:**
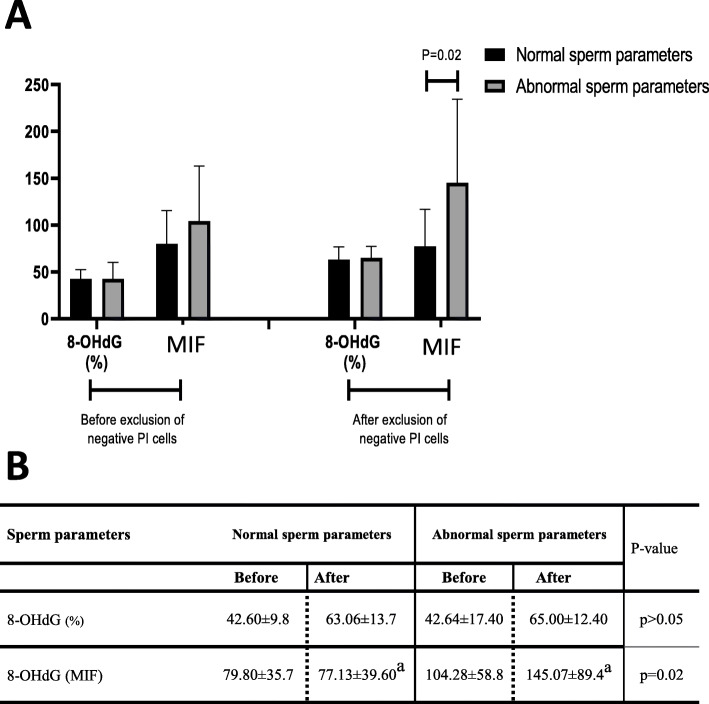
8-OHdG detection. **A/** Comparison of sperm parameters for individuals with normal and abnormal standard sperm parameters (*N* = 13 and *N* = 14, respectively). Data are presented as the mean ± standard deviation. **B**/ Comparison of mean intensity of fluorescence (MIF in arbitrary units) and percentage (%) of 8-OHdG positive cells in individuals with normal and abnormal sperm parameters. Data are presented as mean ± standard deviation. Similar letters indicate significant differences at *p* < 0.05

To investigate whether non-cellular contaminating structures could also influence other types of FC-based spermatozoa analyses, we built up a small subcohort of 14 normozoospermic (normal) samples and 13 samples lying outside of the WHO standard seminal parameters (abnormal) mainly with regard to sperm concentration and motility (see supplementary Figure 1B). This cohort was then analyzed by FC to determine the level of 8-OHdG residues detected in the nucleus of spermatozoa in order to assess oxidative nuclear damage (Fig. 3A). As described earlier in Vorilhon et al., [23] two parameters were monitored by FC, namely the percentage of 8-OHdG-positive sperm cells and the sample mean intensity of fluorescence (MIF). Figure 3 shows that before or after exclusion of PI-negative structures (quadrants Q3 and Q4), there was no statistically significant change in the percentage of 8-OHdG-positive spermatozoa in either the “normal” or “abnormal” subcohort. However, a clear upward trend was recorded since in each cohort (“normal” or “abnormal”) the percentage of 8-OHdG positive cells increased from 42 to > 60%, attesting to the underestimation caused by the presence of structures that FC considers as possible spermatozoa. For the MIF assessment, statistical significance was reached only in the “abnormal” subcohort when MIF values were compared before and after exclusion of M540 bodies (*P* = 0.02, Fig. 3). This is likely explained by the greater presence of heterogeneous M540 bodies in the abnormal samples, some of which emit a certain level of fluorescence.

**Fig. 4:**
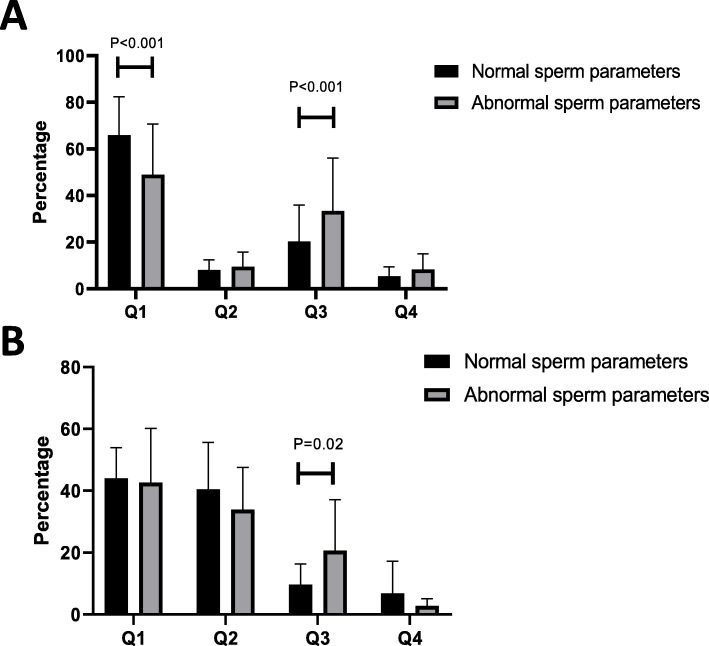
Distribution of events detected in the quadrants of the flow cytometry charts by TUNEL (**A**) and 8-OHdG (**B**) evaluations in normal and abnormal semen samples. Quadrants Q1 to Q4 are as defined in Fig. 1. The data are presented as mean ± standard deviation. The addition of Q1 + Q2 + Q3 + Q4 represents 100% of the events detected. Note that in each case the events recorded in Q3 are always significantly greater in abnormal samples. In addition, as expected, there are significantly fewer negative TUNEL cells in the Q1 quadrant in the abnormal samples. As previously indicated by Vorilhon et al. [23] with regard to evaluation of 8-OHdG, the percentage of 8-OHdG-positive cells alone is insufficient to distinguish normal from abnormal samples (no significant differences in the Q1 and Q2 quadrants). The mean fluorescence intensity of the sample should also be taken into consideration [25]

Figure 4 shows that acellular structures including M540 bodies found in both quadrants Q3 and Q4 (negative for PI staining) of the FC charts accounted for a significant fraction of the FC recorded events ranging grossly from 20 to 40% of the detected events (see Fig. 4A and B). These acellular structures distorted the evaluation, particularly for abnormal semen samples, as the events recorded in quadrant Q3 were significantly more represented in abnormal samples when compared with normal samples.

## Discussion

Flow cytometers, unlike human operators, cannot distinguish between fluorescence-emitting cellular or non-cellular biological structures. To partially circumvent this problem, gating strategies are employed using forward scattering / lateral scattering (FSC/SSC) and size parameters for the cells under analysis. Nevertheless, there are situations where the size criterion is insufficient, especially when there are non-cellular structures of similar size to the cells being monitored in the sample. This is particularly relevant for semen samples (prepared or not) which contain, in addition to the very small sperm cell type, various non-cellular structures, including M540 bodies, so-called because of their reactivity to merocyanine 540 [18–22, 25, 26]. M540 bodies are heterogeneous structures that are devoid of chromatin. This feature provides a means of distinguishing them from spermatozoa since they are not labeled by a DNA intercalating agent such as propidium iodide (PI), as reported by Muratori et al. [25]. These structures have been shown to be more abundant in particular conditions such as testicular dysfunction, including elevated FSH (follicle stimulating hormone) levels, testicular hypotrophy, defective spermatogenesis or increased testicular apoptosis [26]. With regard to the later it was reported that M540 structures were reactive to several apoptotic markers including Fas, p53, Bcl-X and Caspase 3, suggesting that they could be remnants of testicular apoptotic cells [19, 20, 27]. In clinical terms, the abundance of these structures in semen correlates with male infertility.

Muratori et al., and Ribeiro et al., [16, 21] proposed that because of their size and increased abundance in pathological semen samples (confirmed by the present work), the presence of M540 bodies could hamper the accuracy of evaluated parameters when FC was used to assess spermatozoa integrity, particularly if the semen samples were not fractionated prior to evalution. However, this was not widely publicized and some controversy subsisted [24] which led the andrology community to continue using FC to monitor spermatozoa structural and functional defects without accounting for this potentially important observation. During recent years considerable global effort has focused on extending and improving the approaches taken by clinicians to evaluate the male partner of an infertile couple. Thus, we felt it was extremely important to verify the accuracy of our FC-assisted spermatozoa evaluations in the clinical setting. To this end we performed a comparison of FC data obtained in our clinical center in the presence or absence of M540 bodies. Data was obtained pertaining to sperm DNA fragmentation (SDF) and sperm DNA oxidation (SDO) as part of our standard evaluation of the infertile male. In order to exclude the M540 structures from the sample under analysis, we introduced a supplementary step to each FC protocol (SDF or SDO): propidium iodide (PI) was used following the rationale that unlike fixed dead spermatozoa, M540 bodies would not react with PI as they contain fragmented DNA in which PI could not intercalate [28, 29]. Incorporation of the PI step allowed us to exclude all PI-negative structures from the analysis including M540 bodies within the same FFC/SSC region (Q3 and Q4 quadrants in the FC charts). We noticed that the events detected by FC in the Q3 and Q4 quadrants were considerable, particularly for abnormal semen samples. The majority of these events concerned the Q3 quadrant in which the acellular structures that were unreactive to either PI or TUNEL were found. This was to be expected given that these structures are devoid of intact DNA material. Also surprising, although to a lesser extent, were the events detected by FC in the Q4 quadrant corresponding to acellular structures devoid of DNA, but nevertheless TUNEL-positive. Ribeiro et al. believe that two types of PI cells exist in a semen sample and call them “PI-bright” and “PI-dim”. They proposed that PI-dim are spermatozoa whose nucleus is so condensed that PI cannot penetrate, thus appearing as PI-negative (16).

By removing the contribution of the Q3 and Q4 quadrants from the FC-assisted analyses, we observed that significance was reached for both assays when the normal cohort was compared with the abnormal cohort. This was in contrast to results obtained in the presence of the PI-negative structures or when Q3 and Q4 quadrants were not excluded. For the 8-OHdG assay in which two FC parameters were monitored (i.e. the percentage of 8-OHdG-positive cells in the sample and the mean intensity of sample fluorescence), significance was reached only for the second parameter. This was expected, as we had already demonstrated that the percentage of 8-OHdG-positive cells was not a sufficiently discriminating parameter on its own and that it should be associated with the mean fluorescence intensity of the sample [23]. Interestingly, when using the SCSA assay in parallel, which was also performed with FC, no significant difference was observed with or without the M540 structures. This is probably related to the very different nature of these tests. This reinforces the idea that the SCSA is one of the best tests to use to assess sperm nuclear integrity via FC.

Our findings are in complete agreement with the earlier demonstration by the Italian research group [19–21, 30] and show that FC-assisted evaluations of unfractionated semen samples are biased by the presence of non-cellular structures such as M540 bodies. If, FC-data are not corrected by excluding these PI-negative components, they deliver a false view of the true integrity of the sperm population. This is of utmost importance as a reliable and precise evaluation to guide clinicians with their therapeutic options. Although in comparison to classical microscopic approaches FC provides a better estimate of the monitored parameters because of non-subjectivity and the fact that a large number of cells can be analyzed, it is important to ensure that the samples are free from contaminating structures such as the M540 bodies highlighted here. In the case of sperm samples, it is critical to eliminate these contaminants as they are known to be more represented in pathological cases of infertility [19–21] where the assessment accuracy may be of paramount importance to clinical management. We provide confirmation of the findings originally published by the University of Firenze research group and strongly recommend that PI staining should routinely accompany FC-assisted evaluation of sperm DNA/nuclear integrity for a more accurate evaluation.

Overall, the effect of sperm DNA fragmentation on fertility and ART outcomes has become a hot topic in the field of andrology. Many publications recommend the evaluation of sperm DNA fragmentation as part of routine semen analysis, while other reports question the benefits of such evaluation. Despite the very recent WHO position (WHO2021) that the classic “baseline semen examination” could be advantageously complemented by an “extended examination” that includes sperm DNA/nuclear integrity, there remain conceptual and practical problems that prevent its widespread use in the clinical community. The lack of consistency and concordance between the different tests used, the subjectivity of the operators, and the inter-clinical variations prevent the community from adopting a consensual approach. This leads the WHO to propose that each laboratory should define its own threshold value for nuclear sperm integrity. We believe that one of the reasons for this inconsistent picture may be due in part to the presence of these M540 bodies which vary between individuals. If this is taken into account, it may help reducing the inter- and intra-variability that currently clouds the relevance of sperm DNA nuclear integrity assessment.

## Conclusions

Despite the fact that non-cellular structures such as M540 bodies exist in semen and are present at a higher density in the semen of men with severely compromised spermatogenesis, clinicians and researchers studying sperm nuclear integrity by flow cytometry continue to ignore this issue. This poses the question of the pertinence and clinical value of the underestimated FC-assisted evaluation of sperm nuclear integrity data. We believe that dismissing this topic undermines the value of FC-assisted monitoring of unfractionated sperm samples. Furthermore, it contributes to diminishing the confidence the community should have in the emergence of these new assays in the clinical armamentarium to better deal with infertile couples and the issues associated with ART using spermatozoa with paternal DNA/nuclear damage. The underestimated FC-assisted evaluation of sperm DNA integrity may explain the discrepancies that some authors have found when comparing their data with conventional fluorescent microscopic assessment, leading to some confusion and reluctance about the added value of these assessments [15, 31]. In recent years the European Society of Human Reproduction and Embryology (ESHRE) has placed strong emphasis on the importance of better evaluating the integrity of the paternal nucleus, particularly in terms of the degree of fragmentation, which was convincingly associated with early pregnancy loss [32–34]. There is also growing concern in the community that any paternal DNA damage (breaks and base oxidation) that escapes repair by the oocyte is the gateway to the transfer of de novo mutations to future generations [35]. Therefore, it is important to implement the most precise evaluation of the true level of sperm DNA damage.

## Supplementary Information

**Additional file 1.**

## Data Availability

All data generated or analyzed during this study are provided.

## Abbreviations

8-OHdG: 8-oxo-7,8-dihydro-2′-deoxuguanosine
AO: acridine orange
ART: assisted reproductive technology
DFI: DNA fragmentation index
DNA: deoxyribo nucleic acid
DTT: dithiothreitol
ESHRE: European Society for Human Reproduction and Embryo
FITC: fluorescein isothiocynanate
FC: flow cytometry
FM: fluorescence microscopy
FSC/SSC: forward scatter/sidewards scatter
HDS: high DNA stainability
ICSI: intracytoplasmic sperm injection
M540: merocyanine 540
MIF: Mean intensity of fluorescence
PBS: phosphate buffer saline
PI: propidium iodide
SCD: sperm chromatin dispersion
SCSA: sperm chromatin structure assay
SD: standard deviation
SDF: sperm DNA fragmentation
SDO: sperm DNA oxidation
Tdt: termainal transferase
TNE: Tris-NaCl-EDTA
TUNEL: terminal deoxynucleotidyl transferase-mediated deoxyuridine triphosphate-biotin nick end labeling
WHO: world health organization
