# Supplementary material for: Apoptotic M540 bodies present in human semen interfere with flow cytometry-assisted assessment of sperm DNA fragmentation and oxidation
Source: Basic Clin Androl. 2021 Oct 21;31:23. doi: 10.1186/s12610-021-00143-7 (PMC8529777; doi:10.1186/s12610-021-00143-7)
Supplement: Supplementary file 1 — Additional file 1. [file 12610_2021_143_MOESM1_ESM.pdf]

A

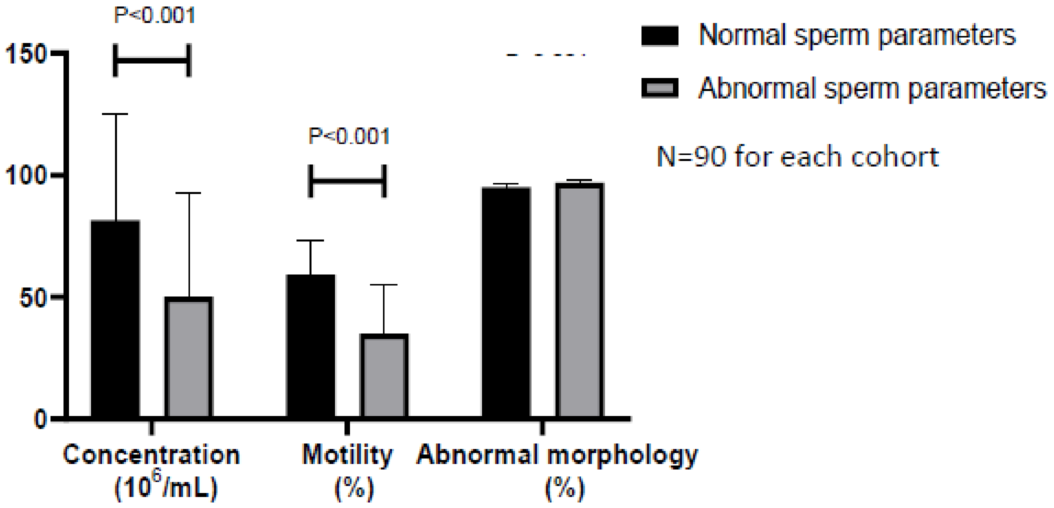

B

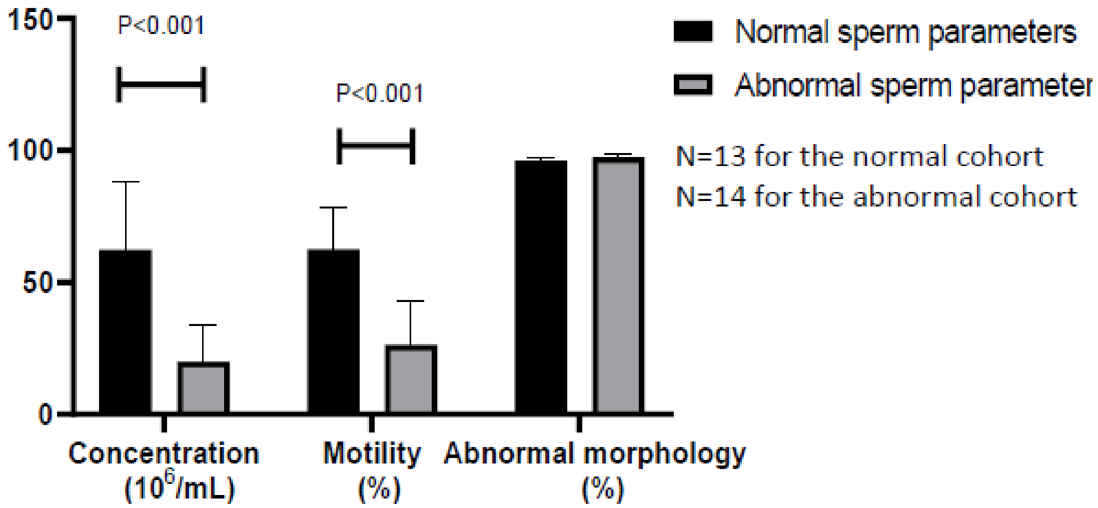

Supplementary Figure 1:

Routine basic semen analysis (WHO criteria : spermatozoa concentrations in 10^6 cells/ml, motility, morphology in the normal and abnormal cohorts (N= 180 total) analyzed for sperm DNA frgamentation by the TUNEL assay **(A)** and for sperm DNA oxidation **(B)** by the 8-OHdG assay (N= 27 total). Data are presented as the mean ± standard deviation.
